# Instability of 8E5 calibration standard revealed by digital PCR risks inaccurate quantification of HIV DNA in clinical samples by qPCR

**DOI:** 10.1038/s41598-017-01221-5

**Published:** 2017-04-26

**Authors:** Eloise Busby, Alexandra S. Whale, R. Bridget Ferns, Paul R. Grant, Gary Morley, Jonathan Campbell, Carole A. Foy, Eleni Nastouli, Jim F. Huggett, Jeremy A. Garson

**Affiliations:** 1 Molecular and Cell Biology Team, LGC, Teddington, UK; 2 Department of Infection, Division of Infection and Immunity, University College London, London, UK; 3 Department of Clinical Virology, University College London Hospital NHS Foundation Trust, and the UCL/UCLH NIHR Biomedical Research Centre, London, UK; 4 Department of Population Policy and Practice, UCL GOS Institute of Child Health, London, UK; 5 School of Biosciences & Medicine, Faculty of Health & Medical Science, University of Surrey, Guildford, GU2 7XH UK; 6 National Transfusion Microbiology Laboratories, NHS Blood and Transplant, Colindale, London, UK

## Abstract

Establishing a cure for HIV is hindered by the persistence of latently infected cells which constitute the viral reservoir. Real-time qPCR, used for quantification of this reservoir by measuring HIV DNA, requires external calibration; a common choice of calibrator is the 8E5 cell line, which is assumed to be stable and to contain one HIV provirus per cell. In contrast, digital PCR requires no external calibration and potentially provides ‘absolute’ quantification. We compared the performance of qPCR and dPCR in quantifying HIV DNA in 18 patient samples. HIV DNA was detected in 18 by qPCR and in 15 by dPCR, the difference being due to the smaller sample volume analysed by dPCR. There was good quantitative correlation (R^2^ = 0.86) between the techniques but on average dPCR values were only 60% of qPCR values. Surprisingly, investigation revealed that this discrepancy was due to loss of HIV DNA from the 8E5 cell calibrant. 8E5 extracts from two other sources were also shown to have significantly less than one HIV DNA copy per cell and progressive loss of HIV from 8E5 cells during culture was demonstrated. We therefore suggest that the copy number of HIV in 8E5 extracts be established by dPCR prior to use as calibrator.

## Introduction

HIV continues to be a major issue for global health, with approximately 36.7 million people living with HIV at the end of 2014 and about 2 million individuals becoming infected each year (WHO 2015). Despite the advent of effective combination antiretroviral therapy (cART), establishing a cure is hindered by the persistence of latently infected host cells, even in the absence of detectable plasma viraemia^1, 2^. These cells, usually CD4+ resting T cells, constitute the viral reservoir^3^ and have the potential to release progeny virions, therefore being responsible for viral rebound after discontinuation of therapy^4, 5^. With the advent of novel strategies for HIV cure that include latency reversing agents^6, 7^ accurate and robust methods are required for measurement and monitoring of the latent reservoir^8^.

A routine method for quantification of HIV RNA viral load, real-time quantitative PCR (qPCR), is also increasingly being used for measuring HIV DNA associated with the viral reservoir^9^. qPCR requires calibration and for this to be reproducible it is essential that the calibrator must be stable when shared between laboratories. A popular choice of calibrator for quantifying HIV DNA by qPCR is 8E5 (ATCC® CRL-8993)^10–17^, a lymphoblastic leukaemia cell line which has been reported by several studies to contain one integrated HIV genome per cell^12, 18, 19^.

Digital PCR (dPCR) is a more recently developed method that offers absolute quantification^20^. It has been used to value assign a variety of qPCR calibrators, including those for BCR-ABL^21^ and Mycobacterium tuberculosis^22^. dPCR has also been used in the direct quantification of HIV DNA from patients in a number of studies^23–27^ and unlike qPCR has the advantage of not requiring an external calibration standard. However, false positives and issues surrounding threshold determination have been reported to limit the usefulness of dPCR when employed for the most sensitive measurements of HIV DNA^28^. In this study we investigated the application of dPCR instruments in the context of HIV DNA measurement, both for comparison with qPCR analysis of patient samples and as a method for value assigning 8E5 calibration standards from three different sources.

## Methods

### Patient samples and 8E5 cell calibration standards

Anonymised peripheral blood mononuclear cell samples (PBMC) were obtained from HIV-positive individuals receiving antiretroviral therapy as part of a recently published clinical trial^29^ comparing Short Cycle Therapy (SCT) with continuous antiretroviral therapy. The study had received appropriate ethical committee approval.

Aliquots of DNA extracted from the 8E5 cell line^19^ were obtained from three separate institutions and designated Standard 1, Standard 2 and Standard 3. Standard 1 had been used for clinical research on HIV DNA levels; Standard 2 had been used in research as a source of HIV RNA; Standard 3 was a freshly obtained 8E5 cell culture from the American Type Culture Collection (ATCC® CRL-8993™) distributed by LGC, Teddington, UK. The passage numbers of the 8E5 cells from which Standard 1 and Standard 2 were obtained were unknown.

### Culture of 8E5 cells (Standard 3)

Briefly, one vial of 8E5 cells (ATCC® CRL-8993™) was taken from liquid nitrogen and thawed at 37 °C for 1–2 minutes. 500 µL of cells was removed from the vial for culture and the remaining 300 µL (approximately 2.4 × 10^6^ cells) retained for DNA extraction as passage 0 (P0). The full culture methodology is described in Supplementary Information. Following the single initial flask (designated passage 1), successive passages were maintained in triplicate (three separate flasks for passages 2, 3 and 4). During each passage cells were taken, pelleted and stored at −80 °C prior to DNA extraction.

### DNA extraction

120 µL of each PBMC sample was lysed in 120 µL ATL buffer and the nucleic acid extracted on the QIAsymphony platform (Qiagen) using the DSP Virus/Pathogen Mini Kit (Qiagen) according to manufacturer’s protocols. Extracts were eluted in 60 µL and stored at −20 °C prior to analysis. DNA was extracted from the 8E5 Standard 3 cell pellets from each culture passage using the QIAamp DNA Blood Mini Kit (Qiagen). Supplemental to the manufacturer’s protocol, extracts were treated with 4 µL of RNase A (Qiagen) prior to the addition of lysis buffer. Final elution volume was 200 µL in buffer AE.

### PCR assay design and primer sequences

Sequence information for the primers and probes used in the study is given in Table S1 of Supplementary Information. All PCR assays were performed in duplex format (i.e. two PCRs in the same reaction tube) consisting of one human reference gene assay (either pyruvate dehydrogenase, PDH or RNase P) and one assay specific for HIV-1. The HIV LTR-*gag* assay was designed to span a highly conserved region of the LTR-*gag* junction to allow amplification of a single Long Terminal Repeat.

### qPCR analysis of clinical samples

qPCR analysis of 18 PBMC sample extracts was performed using an Applied Biosystems® 7500 Real-Time PCR System. Experiments were implemented in accordance with the MIQE guidelines^30^ (Table S2, Supplementary Information). To prepare a qPCR calibration curve consisting of ~50,000 to ~5 HIV DNA copies per reaction (assuming 1 HIV DNA copy per 8E5 cell), DNA extracted from the 8E5 cell line Standard 1, (DNA concentration initially established by Qubit fluorometric quantitation; ThermoFisher Scientific Inc.), was serially diluted using a tenfold dilution series in 5 µg/mL carrier RNA (Qiagen) dissolved in nuclease-free water. Twenty µL of each clinical sample extract (~1.2 µg DNA, equivalent to approximately 200,000 cells) was added to a total reaction volume of 50 µL. Full details of the PDH/HIV LTR-*gag* duplex qPCR assay protocol, cycling parameters and primer/probe sequences are given in Supplementary Information.

### Digital PCR basic protocol

Duplex format dPCR experiments were implemented in accordance with the dMIQE guidelines (Supplementary Information)^31^. Two dPCR instruments were employed during the study; the RainDrop^®^ Digital PCR System (RainDance Technologies) was used to measure the clinical samples and 8E5 extracts, and the QX200™ Droplet Digital™ PCR System (BioRad) was used to measure the 8E5 extracts only. Positive and negative partitions were selected for the RainDrop^®^ and QX200™ manually using ellipse and quadrant gating, respectively, as recommended by the manufacturer using the instruments’ software. Full experimental protocols for both dPCR instruments and details of primers and probes used in the duplex assays are given in Supplementary Information. No template controls (NTCs) were included in all experiments.

### Digital PCR analysis of clinical samples

5 µL (equivalent to approximately 50,000 cells) of the same 18 clinical sample extracts that had previously been analysed by qPCR were analysed using the RainDrop^®^ dPCR platform as described above. The samples were amplified using the PDH/HIV LTR-*gag* duplex assay and NTCs of nuclease-free water were included as controls. The extracts were coded and the operator had no prior knowledge of the qPCR results on the same samples.

### Digital PCR characterisation of 8E5 cells

The 8E5 DNA extracts from Standards 1, 2 and 3 were assessed using the RainDrop^®^ platform with duplex primer sets to PDH/HIV LTR-*gag*, PDH/HIV *pol* and RNase P/HIV LTR-*gag* (Table S1). For the Standard 3 cells all four culture passage extracts and the initial passage zero (P0) extract were analysed using both RainDrop^®^ and QX200™ instruments with the PDH/HIV LTR-*gag* duplex assay.

### Effect of different 8E5 calibrator sources on qPCR analysis of clinical samples

Analysis of an additional seven HIV-positive clinical sample PBMC extracts was performed using qPCR as above. This experiment utilised the three different 8E5 cell Standards 1, 2 and 3 (passage 2) simultaneously as calibrators in the same run. HIV DNA copies were calculated per million cells using either the published quantity of 1 HIV DNA copy per 8E5 cell^12, 18, 19^ for all three different 8E5 sources or alternatively, the quantity determined empirically for the respective 8E5 extracts by dPCR during the present study.

### Data Analysis

Data from dPCR and qPCR experiments were subject to threshold and baseline setting in the relevant instrument software, and were exported as .csv files to be analysed in Microsoft Excel 2010. For dPCR experiments the average number of copies per droplet (λ) was calculated as described previously^32^. dPCR and qPCR analyses of the clinical samples were compared by using a paired t-test on the log transformed HIV DNA copies per million cells. Agreement between methods was investigated using a Bland-Altman analysis and data evaluated for linearity using linear regression.

## Results

### Analysis of clinical samples by qPCR and dPCR

**Figure 1:**
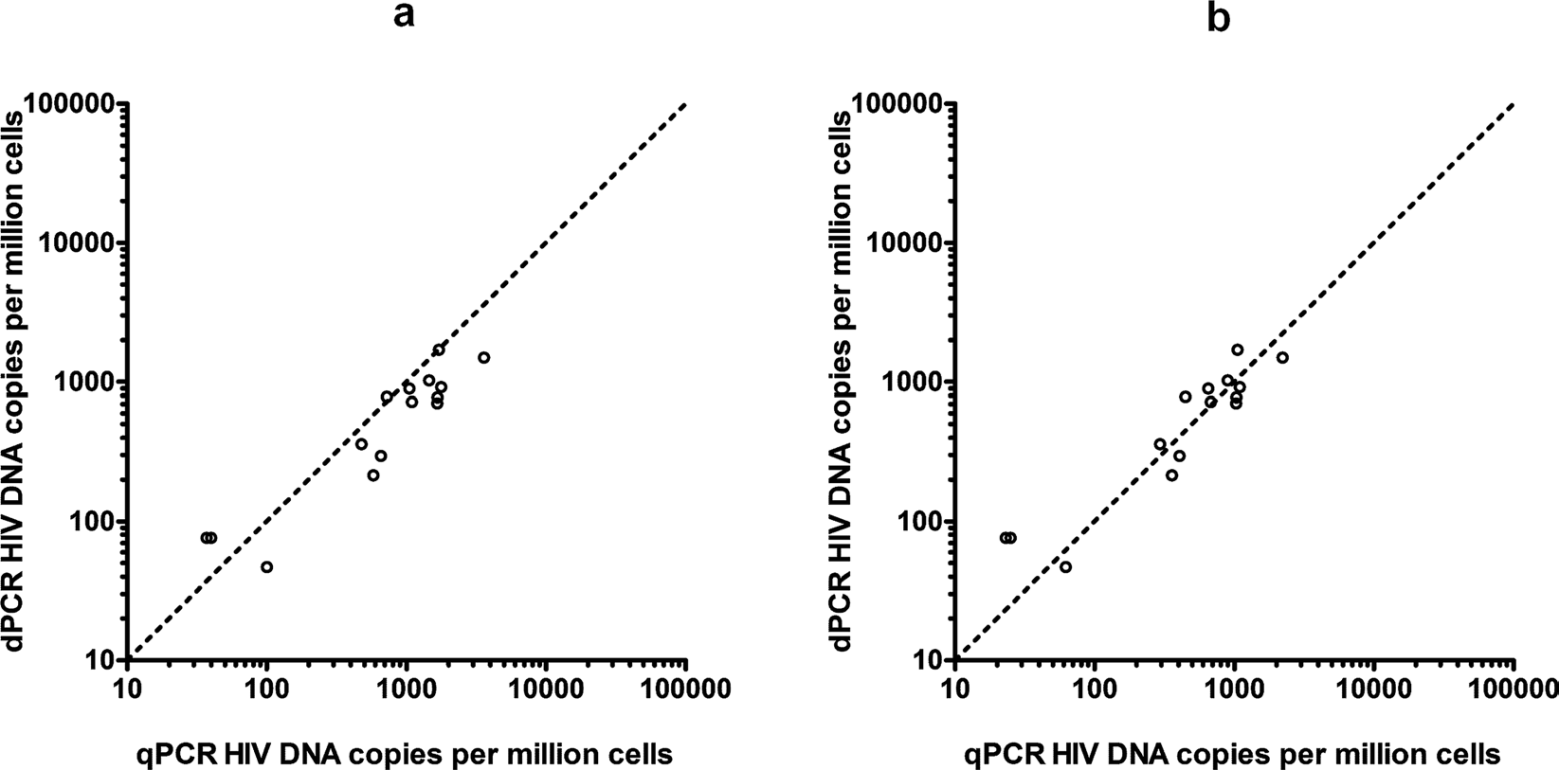
Comparison between dPCR and qPCR results from 18 PBMC samples from HIV-positive patients, expressed as HIV DNA copies per million cells. The three samples in which HIV DNA was not detected by dPCR are not plotted. (**a**) qPCR quantities calculated assuming one HIV DNA copy per 8E5 cell. (**b**) qPCR quantities calculated assuming 0.6 HIV DNA copy per 8E5 cell as determined experimentally by dPCR NB. The dashed line represents equivalence.

18 PBMC DNA samples from HIV-positive patients were analysed by dPCR and qPCR using the PDH/HIV LTR-*gag* duplex assays. HIV DNA was detected in all 18 samples by qPCR but in only 15 samples by dPCR. The three dPCR negative samples were near the lower limit of detection by qPCR (Supplementary Information, Table S3) and were probably undetected by dPCR due to the lower volume of template used (RainDrop^®^ dPCR used ~5 µL whereas the qPCR used 20 µL, an approximately 4 fold greater volume of template). When the HIV DNA copies per million cells were calculated the dPCR and qPCR results correlated well (R^2^ = 0.86), however the dPCR results were on average only ~60% of the qPCR results, a statistically significant difference (p = 0.02) (Fig. 1a). Linear regression on the data generated from Bland-Altman analysis found no evidence of a trend in the observed bias which was independent of HIV DNA concentration (Figure S3). No false positive dPCR results were observed in the NTCs (Supplementary Information, Table S4).

### Discrepancy between qPCR and dPCR due to loss of HIV DNA from 8E5 cells

**Figure 2:**
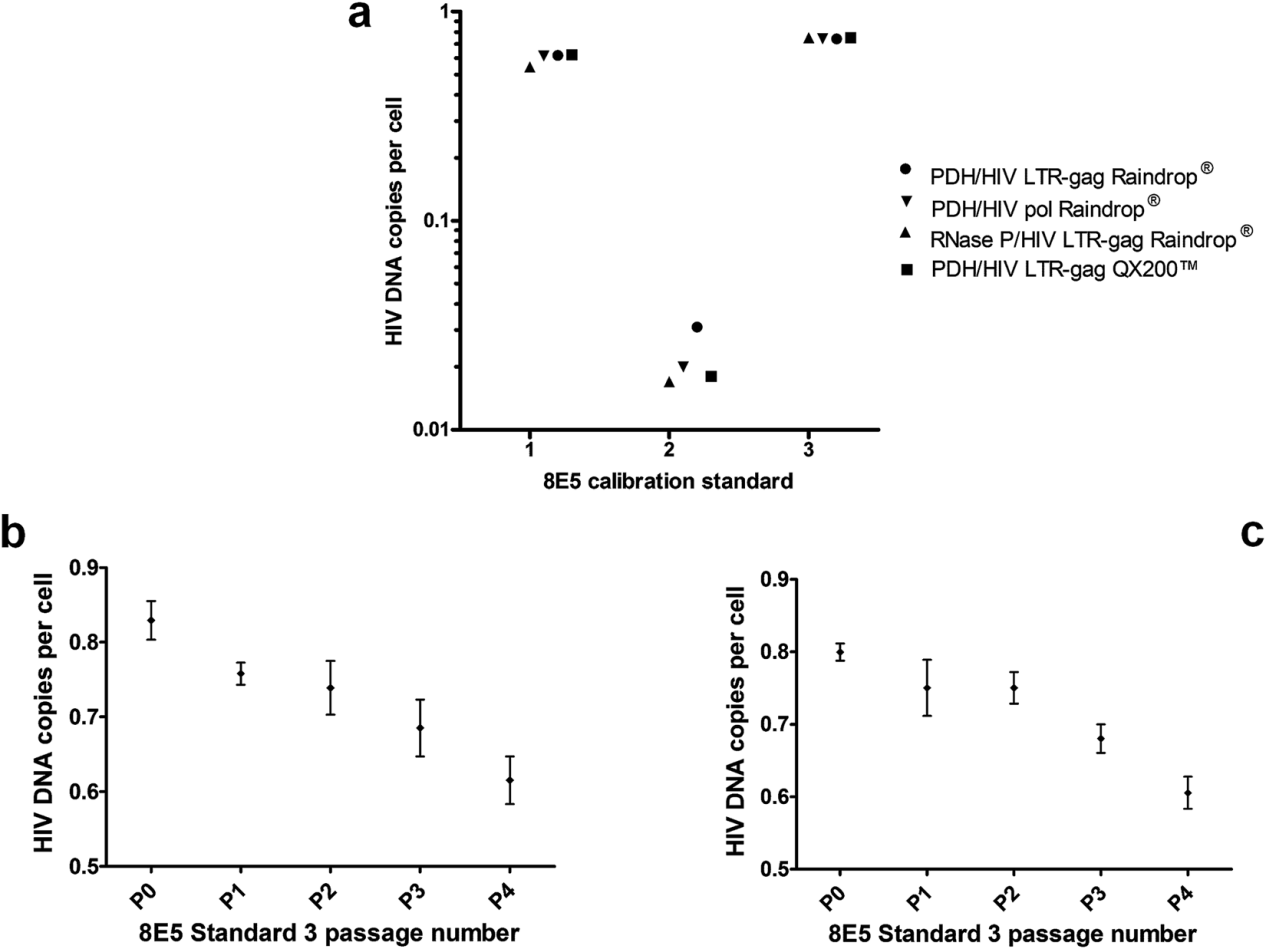
HIV DNA copies per cell calculated for different 8E5 sources. (**a**) Comparison of 8E5 Standards 1, 2 and 3 analysed by dPCR. (**b**) Effect of culture passage on HIV DNA content per cell for 8E5 Standard 3 measured using the RainDrop^®^ dPCR platform (**c**) Effect of culture passage on HIV DNA content per cell for 8E5 Standard 3 measured using the QX200™ dPCR platform. Mean values with standard deviations are plotted.

In order to determine whether the ~60% discrepancy between dPCR and qPCR results might have been due to erroneous calibration of the qPCR, we investigated the calibrator (8E5 Standard 1) that had been used to calibrate the qPCR assay. Surprisingly, RainDrop® dPCR analysis of 8E5 Standard 1 with the PDH/HIV LTR-*gag* duplex assay revealed a ratio of PDH copies to HIV copies of approximately 3.2:1, whereas according to the literature^12, 18, 19^ the expected PDH:HIV ratio should have been exactly 2:1. This surprising finding was confirmed by repeating the dPCR analysis of 8E5 Standard 1 with a different dPCR instrument (QX200™) and with a different region of the HIV genome (*pol*) as PCR target. To exclude the possibility that the unexpected PDH:HIV ratio in 8E5 Standard 1 might have been caused by an increase in the PDH reference gene copy number we repeated the assays using a different human reference gene (RNase P) located on a different chromosome. In all cases the results confirmed that the PDH:HIV ratio in 8E5 Standard 1 was approximately 3.2:1 which is equivalent to approximately 0.6 HIV DNA copies per 8E5 cell. These findings are summarised in Fig. 2a. When the qPCR results on the 18 clinical samples were corrected to take into account the actual HIV DNA content of the 8E5 Standard 1 used as calibrator, the ~60% discrepancy between qPCR and dPCR findings became statistically insignificant (p = 0.41) (Fig. 1b).

To establish whether this loss of HIV DNA from the 8E5 calibrator was unique to the particular source of 8E5 that had been used, we obtained additional aliquots of 8E5 (designated Standard 2 and Standard 3 for the purposes of this study) from two independent institutions. 8E5 DNA extracts from Standard 2 and Standard 3 were analysed with both RainDrop® and QX200™ dPCR instruments by duplex assays using both regions of the HIV genome as target and both human reference genes. Remarkably, the magnitude of the loss of HIV DNA from 8E5 Standard 2 proved to be even greater (~0.02 HIV DNA copies per cell) than for Standard 1. In contrast, the loss of HIV DNA from 8E5 Standard 3 (~0.8 HIV DNA copies per cell) was less marked. The results of this dPCR characterisation of 8E5 Standards 2 and 3 are shown in Fig. 2a.

For the ATCC stock (8E5 Standard 3) five separate culture passages were analysed starting from baseline (P0) to passage 4. One DNA extract representing each culture flask per passage was analysed on RainDrop^®^ and QX200™ dPCR platforms using the PDH/HIV LTR-*gag* duplex assay. The HIV DNA copies were observed to decrease relative to PDH copies with successive passages, equating to a fall in HIV DNA copy number from ~0.8 to ~0.6 copies per cell (Fig. 2b and c). Short Tandem Repeat (STR) analysis was performed by the supplier prior to culture, with the unique DNA profile being concordant with the cell line specification, suggesting proliferation of an additional non related clonal population was unlikely to be the source of this HIV DNA copy number change. No false positive results were observed for either instrument during these comparisons (Table S4).

### Different sources of 8E5 calibrator may generate significant inaccuracies in HIV DNA quantification of clinical samples

**Figure 3:**
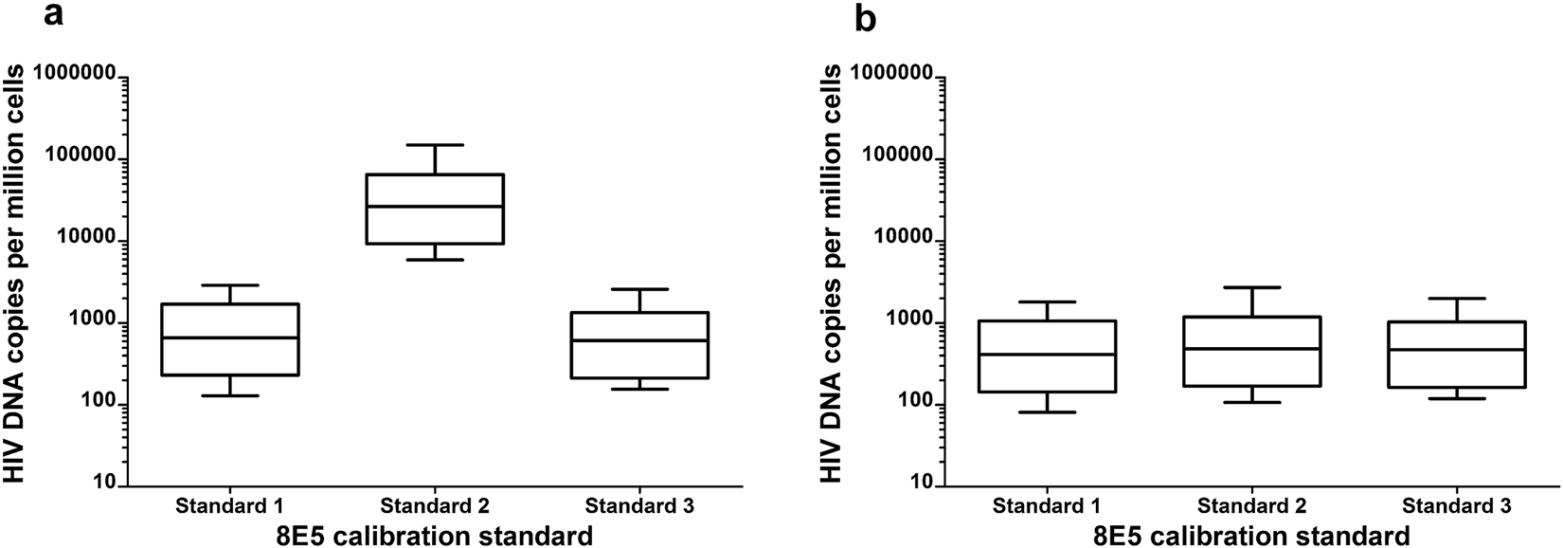
Median HIV DNA copies per million cells (boxplots with interquartile and range) of seven clinical samples assayed in duplicate by qPCR using 8E5 Standards 1, 2 and 3 for calibration. (**a**) Calculated assuming one HIV DNA copy per 8E5 cell for all three Standards. (**b**) Calculated using the actual number of HIV DNA copies per 8E5 cell as determined by dPCR for each of the three Standards.

To assess the effect of using different sources of 8E5 calibration material on the qPCR quantification of HIV DNA in clinical samples, three separate standard curves were constructed from 8E5 Standards 1, 2 and 3 in the same experimental run. Seven additional patient PBMC samples were tested in duplicate by qPCR using the PDH/HIV LTR-*gag* duplex assay and the means (expressed in HIV DNA copies per million cells) calculated for each sample (Fig. 3). When the previously reported one HIV DNA copy per 8E5 cell was assumed for all three Standards, the values calculated using 8E5 Standard 2 as calibrator were approximately 45 times higher than those calculated using the 8E5 Standards 1 and 3 which agreed with each other (Fig. 3a). When the dPCR derived values of HIV DNA copies per 8E5 cell were applied to the respective 8E5 Standard 1, 2 and 3 extracts the results with all three sources of 8E5 calibrator became concordant (Fig. 3b).

## Discussion

Detection of total cellular HIV DNA, comprising integrated proviral DNA and unintegrated forms such as LTR circles, offers a means of monitoring the latent viral reservoir in the absence of circulating HIV RNA^5^. However, it should be noted that HIV DNA assays are unable to differentiate between replication competent and incompetent HIV genomes and therefore do not actually measure the *functional* viral reservoir, which is most directly assessed by viral outgrowth assays^24^. Notwithstanding these reservations, HIV DNA assays have been widely used as an alternative to viral outgrowth assays because the latter are disadvantaged by being relatively expensive, labour intensive, technically demanding and requiring large amounts of blood. There is data supporting the use of HIV DNA assays and reports indicating a correlation between HIV DNA levels and clinically important parameters such as disease progression, post-treatment virological control and time to viral rebound on stopping cART^5^. qPCR is a widely used method for measuring total cellular HIV DNA^9^ as it is a versatile technique that is already well established for HIV RNA viral load measurement. It is comparatively inexpensive and readily scalable both in terms of reaction volume and throughput.

More recently, digital PCR has also been applied for HIV DNA measurement with some success but concerns have been raised regarding its sensitivity^28^. In this study we aimed to compare qPCR with dPCR for measuring total cellular HIV DNA in clinical samples and attempted to explain why discrepancies between the techniques may have occurred. We found that the results were broadly comparable, but that dPCR had reduced sensitivity related to the lower sample volume protocol employed. We did not observe the false positive dPCR results reported by others^28^ with either RainDrop^®^ or QX200™ platforms (Table S4) and so did not have the challenge associated with setting thresholds to omit false positives. This demonstrates that dPCR could be effective as an alternative to qPCR for measurement of HIV DNA in patient samples if adequate sample volumes are used and strict contamination control measures maintained.

While dPCR may offer a powerful alternative ‘absolute’ method to qPCR for research use, the fact that the latter technique is so well established means it is likely to remain the method of choice for most clinical analyses of HIV DNA in the short term at least. However, this study has demonstrated that dPCR has an important role in improving qPCR accuracy and reproducibility by characterising and value assigning the calibration materials used for qPCR quantitation; we identified that qPCR overestimated the amount of HIV DNA per million cells due to unexpected instability of the 8E5 cell calibrator. The 8E5 cell line has been repeatedly reported and assumed to contain one HIV DNA proviral genome per cell^12, 18, 19^ but our findings suggest that this assumption is unsafe and that different batches of 8E5 may contain different amounts of HIV DNA per cell (varying in this study from ~0.02 to ~0.8 copies per cell).

To determine the HIV DNA copy number in the master stock and investigate the effect of culture on HIV DNA copies, a fresh culture was obtained from ATCC and serially passaged four times. This experiment demonstrated that HIV DNA copies were being lost in culture with serial passage (Fig. 2b and c). Coincidentally, during the preparation of the present manuscript, a study by Wilburn and colleagues was published, also raising concerns over the use of 8E5 for calibrating HIV DNA assays^33^. Wilburn’s study, based on fluorescent *in situ* hybridisation (FISH) and flow cytometry also concluded that, contrary to expectation, deletion of the HIV proviral genome could occur during culture of 8E5 cells and that different batches of 8E5 cells could contain dramatically varying numbers of cells lacking viral genomes. The mechanism of HIV DNA loss is unclear but it may be relevant that the provirus in 8E5 cells is inserted at 13q14-q21 which contains common fragile sites^18^ and could therefore render 8E5 susceptible to proviral loss through genomic instability.

Although qPCR is applied routinely in clinical virology, for the method to be reproducible it is widely recognised that reference materials are needed^34^ from which calibration standards can be derived. Reference materials do not currently exist for HIV DNA measurement, however the 8E5 cell line, with a reported single HIV DNA copy per cell, has been widely used as a calibrator over many years^10–17^. We demonstrate here that using the 8E5 cell line and assuming one HIV DNA copy per cell could lead to inaccuracies which could in turn result in misleading quantitative estimates of the HIV reservoir. Although 8E5 is commonly employed for calibration of HIV DNA qPCR assays, alternative calibrators such as the U1 cell line and HIV plasmids have been used in some studies^23, 27^. Bias of the type described here with 8E5 calibration has not to our knowledge been reported in studies that have utilised these alternatives, however the dPCR approach that we describe can also be used to determine the HIV content of different calibrators.

While we have identified this potential problem and demonstrated the significant bias that may ensue (Fig. 3a) we have also demonstrated how dPCR can be used to rectify any bias and harmonise the quantitative findings from 8E5 sources containing different quantities of HIV DNA (Fig. 3b). It would seem prudent to recommend that laboratories embarking on new quantitative studies into HIV DNA using qPCR obtain a fresh stock of 8E5 or other chosen calibrator and establish its actual HIV DNA content empirically using dPCR. Previous studies that may have used 8E5 with potentially varying HIV DNA quantities can apply the dPCR methods described here to determine the HIV DNA content of the batch used and, if necessary, recalculate their findings based on the new value assignment.

## Electronic supplementary material

Supplementary information
