# Supplementary material for: Instability of 8E5 calibration standard revealed by digital PCR risks inaccurate quantification of HIV DNA in clinical samples by qPCR
Source: Sci Rep. 2017 Apr 26;7:1209. doi: 10.1038/s41598-017-01221-5 (PMC5430807; doi:10.1038/s41598-017-01221-5)
Supplement: Supplementary file 1 — Supplementary information [file 41598_2017_1221_MOESM1_ESM.doc]

**Instability of 8E5 calibration standard revealed by digital PCR risks inaccurate quantification of HIV DNA in clinical samples by qPCR**

## SUPPLEMENTARY INFORMATION

Eloise Busby^1^, Alexandra S. Whale^1^, R. Bridget Ferns^2^, Paul R. Grant^3^, Gary Morley^1^, Jonathan Campbell^1^, Carole A. Foy^1^, Eleni Nastouli^3,4^, ^#^Jim F. Huggett^1, 5^,*, ^#^Jeremy A. Garson^2, 6^,*

*^1^Molecular and Cell Biology Team, LGC, Teddington, UK*

*^2^ Department of Infection, Division of Infection and Immunity, University College London, London, UK*

*^3^ Department of Clinical Virology, University College London Hospital NHS Foundation Trust, and the UCL/UCLH NIHR Biomedical Research Centre, London, UK*

*^4^Department of Population Policy and Practice, UCL GOS Institute of Child Health, London, UK*

*^5^School of Biosciences & Medicine, Faculty of Health & Medical Science, University of Surrey, Guildford, GU2 7XH, UK*

*^6^National Transfusion Microbiology Laboratories, NHS Blood and Transplant, Colindale, London, UK*

**Correspondence should be addressed to* [*jim.huggett@lgcgroup.com*](mailto:jim.huggett@lgcgroup.com) *or* [*j.garson@ucl.ac.uk*](mailto:j.garson@ucl.ac.uk)*.*

*^#^JH and JAG contributed equally to this work*

Table of Contents

[**SUPPLEMENTARY INFORMATION 1**](#__RefHeading___Toc472340062)

[Supplementary Methods 3](#__RefHeading___Toc472340063)

[1. Culture of 8E5 Standard 3cells 3](#__RefHeading___Toc472340064)

[2. Primer/probe sequences 4](#__RefHeading___Toc472340065)

[3. real time and digital PCR MIQE checklist 5](#__RefHeading___Toc472340066)

[4. qPCR/digital PCR experimental protocols 9](#__RefHeading___Toc472340067)

[Supplementary results 11](#__RefHeading___Toc472340068)

[5. Number of HIV DNA copies measured in 18 clinical samples using qPCR and dPCR 11](#__RefHeading___Toc472340069)

[6. Assessing dPCR false positive detection of HIV DNA 12](#__RefHeading___Toc472340070)

[7. Digital PCR analysis of clinical samples 14](#__RefHeading___Toc472340071)

[8. Bland-Altman analysis comparing dPCR with qPCR 15](#__RefHeading___Toc472340072)

[9. Probit analysis of qPCR HIV assay 16](#__RefHeading___Toc472340073)

## Supplementary Methods

## 1. Culture of 8E5 Standard 3cells

The ‘Standard 3’ 8E5 cells were cultured in growth medium that contained RPMI 1640 (ATCC ® 30-2001™) plus 10% foetal bovine serum (ATCC ® 30-2020™) at 37°C in the presence of 5% CO_2_, as recommended by ATCC. A batch suspension culture was maintained between 2.0x10^5^ and 1.0x10^6^ cells/ mL for four successive passages by centrifugation at 900 rpm, and subsequent resuspension in growth medium for stationary incubation in T25 and T75 culture flasks (Corning). Cells were counted using a disposable haemocytometer. Cell pellets representing each passage (passage 1 to 4, plus the initial inoculum) were obtained by centrifugation at 13,500 rpm for 1 minute. The pellets were estimated to contain between 1.0x10^6^ and 4.0x10^6^ cells/ mL, and were stored at -80°C for approximately 1 month prior to DNA extraction.

## 2. Primer/probe sequences

Table S1. Details of primers and probes used in the study

| **Assay target** | **Description** | **NCBI accession number** | **Name** | **5’ to 3’** | **qPCR modifications (5’- 3’)** | **dPCR modifications (5’- 3’)** | **qPCR [Final] (μM)** | **dPCR [Final] (μM)** | **Source** |
| --- | --- | --- | --- | --- | --- | --- | --- | --- | --- |
| Pyruvate Dehydrogenase (PDH) | Reference gene | NG_016860.1 | Forward | TGA AAG TTA TAC AAA ATT GAG GTC ACT GTT | - | - | 0.1 | 0.9 | Present study |
|  |  |  | Reverse | TCC ACA GCC CTC GAC TAA CC | - | - | 0.1 | 0.9 |  |
|  |  |  | Probe (qPCR) | CCC CCA GAT ACA CTT AAG GGA TCA ACT CTT AAT TGT | JOE-TAMRA | - | 0.1 | - |  |
|  |  |  | Probe (dPCR) | CCC CCA GAT ACA CTT AAG GGA | - | VIC-NFQ1 MGB | - | 0.2 |  |
| RNase P (RNase P) | Reference gene | NC_000014.8 | Forward | GCG GAG GGA AGC TCA TCA G | - | - | - | 0.9 | [^1^](#_ENREF_1) |
|  |  |  | Reverse | GGA CAT GGG AGT GGA GTG ACA | - | - | - | 0.9 |  |
|  |  |  | Probe | CAC GAG CTG AGT GCG | - | VIC-NFQ1 MGB | - | 0.2 |  |
| Long Terminal Repeat – gag junction of Human Immunodeficiency Virus-1 (HIV LTR-*gag*) | HIV-1 specific | K03455.1 | Forward | GCC TCA ATA AAG CTT GCC TTG A | - | - | 0.2 | 0.9 | Present study |
|  |  |  | Reverse | GGC GCC ACT GCT AGA GAT TTT | - | - | 0.2 | 0.9 |  |
|  |  |  | Probe | TGT GAC TCT GGT AAC TAG AGA TCC CTC AGA C | FAM-TAMRA | FAM-BHQ1 | 0.1 | 0.2 |  |
| HIV *pol* | HIV-1 specific | K03455.1 | Forward | GCA CTT TAA ATT TTC CCA TTA GTC CTA | - | - | - | 0.9 | [^2^](#_ENREF_2) |
|  |  |  | Reverse | CAA ATT TCT ACT AAT GCT TTT ATT TTT TC | - | - | - | 0.9 |  |
|  |  |  | Probe | AAG CCA GGA ATG GAT GGC C | - | FAM-BHQ1 | - | 0.2 |  |

## 3. real time and digital PCR MIQE checklist

| Table S2. MIQE checklist for authors, reviewers and editors.  *All essential information (E) must be submitted with the manuscript. Desirable information (D) should be submitted if possible.* |
| --- |

| **ITEM TO CHECK** | **IMPORTANCE** | **Comments** |
| --- | --- | --- |
| **EXPERIMENTAL DESIGN** |  |  |
| Definition of experimental and control groups | **E** | Materials and Methods |
| Number within each group | **E** | Materials and Methods;  Supplementary Information section 6 |
| Assay carried out by core lab or investigator's lab? | D | Investigator’s lab |
| Power analysis | D |  |
| Acknowledgement of authors' contributions | D |  |
| **SAMPLE** |  |  |
| Description | **E** | Materials and Methods |
| Volume or mass of sample processed | **E** | 120µL sample extracted. 1/3^rd^ of extract used for each qPCR |
| Microdissection or macrodissection | **E** | N/A |
| Processing procedure | **E** | N/A |
| If frozen - how and how quickly? | **E** | N/A |
| If fixed - with what, how quickly? | **E** | N/A |
| Sample storage conditions and duration (especially for FFPE samples) | **E** | PBMC storage at -80°C for several months and shipping on dry ice |
| **NUCLEIC ACID EXTRACTION** |  |  |
| Procedure and/or instrumentation | **E** | Materials and Methods |
| Name of kit and details of any modifications | **E** | Materials and Methods |
| Manufacturer of reagents used and catalogue number | D |  |
| Details of DNase or RNAse treatment | **E** | Materials and Methods |
| Contamination assessment (DNA or RNA) | **E** | DNA extraction from 8E5 cells: Blank extraction controls (i.e. no sample input) were included in parallel with the samples to assess cross-contamination during extraction. |
| Nucleic acid quantification: Instrument and method | **E** | Qubit 2.0 BR dsDNA (Thermo Scientific) |
| Purity (A260/A280) | D |  |
| Yield | D |  |
| Electrophoresis traces | D |  |
| Quality/integrity-instrument/method; e.g. RIN/RQI and trace or 3’:5’ | **E** | N/A |
| Template structural information | **E** | Available on request |
| Template modification (digestion, sonication, pre-amplification etc.) | **E** | N/A |
| Template treatment (initial heating or chemical denaturation) | **E** | double stranded DNA |
| Inhibition dilution or spike; Cq dilutions | **E** | dPCR: Spiking negative samples with synthetic HIV DNA revealed no evidence of inhibition |
| Storage of nucleic acid: temperature, concentration, duration, buffer | **E** | All nucleic acid was stored at -20°C in either TE or buffer AE (Qiagen). |
| **REVERSE TRANSCRIPTION (If necessary)** |  |  |
| cDNA priming method/oligonucleotide + concentration | **E** | N/A |
| One or two step protocol | **E** | N/A |
| Reaction volume (for two step reverse transcription reaction) | D |  |
| Detailed reaction components and conditions | **E** | N/A |
| Amount of RNA used per reaction | **E** | N/A |
| Reverse transcriptase and concentration | **E** | N/A |
| Temperature and time | **E** | N/A |
| RT efficiency | D |  |
| Estimated copies/Cq values measured with and without addition of RT* | D |  |
| Manufacturer of reagents used and catalogue number | D |  |
| Storage of cDNA: temperature, concentration, duration, buffer | D |  |
| **qPCR/dPCR TARGET INFORMATION** |  |  |
| Sequence accession number | **E** | Supplementary Information section 2. |
| Location of amplicon | D | Materials and Methods, Supplementary Information |
| Amplicon length | **E** | Available on request. |
| In silico specificity screen (BLAST, etc) | **E** | No off-target sequences identified. |
| Pseudogenes, retropseudogenes or other homologs? | D |  |
| Sequence alignment | D |  |
| Secondary structure analysis of amplicon and GC content | D |  |
| Location of each primer by exon or intron (if applicable) | **E** | N/A |
| Where appropriate, which splice variants are targeted? | **E** | N/A |
| qPCR: If multiplex, efficiency and LOD* of each assay. | **E** | LOD approximately 15 HIV DNA copies /million cells. HIV PCR efficiency ~93%; PDH PCR efficiency ~93% |
| **qPCR/dPCR OLIGONUCLEOTIDES** |  |  |
| Primer sequences and/or amplicon context sequence** | **E** | Supplementary Table S1 |
| RTPrimerDB Identification Number | D |  |
| Probe sequences** | D | Supplementary Table S1 |
| Location and identity of any modifications | **E** | Supplementary Table S1 |
| Manufacturer of oligonucleotides | D |  |
| Purification method | D |  |
| **qPCR/dPCR PROTOCOL** |  |  |
| Complete reaction conditions | **E** | Supplementary Information section 4 |
| Reaction volume and amount of RNA/cDNA/DNA | **E** | Supplementary Information section 4 |
| Primer, (probe), Mg++ and dNTP concentrations | **E** | Table S1, Supplementary Information section 4 |
| Polymerase identity and concentration | **E** | N/A (commercial mastermixes used) |
| Buffer/kit Catalogue No and manufacturer | **E** | Supplementary Information section 4 |
| Exact chemical constitution of the buffer | D |  |
| Additives (SYBR Green I, DMSO, etc.) | **E** | N/A |
| Plates/tubes Catalogue No and manufacturer | D |  |
| Complete thermocycling parameters | **E** | Supplementary Information section 4 |
| Reaction setup (manual/robotic) | D | Manual |
| Gravimetric or volumetric dilutions (manual/robotic) | D | Volumetric |
| Master PCR reaction volume prepared | D |  |
| Partition number | **E** | RainDrop® dPCR: up to 1.0x10^7^ partitions |
| Individual partition volume | **E** | RainDrop® dPCR: 0.000005 µL |
| Total volume of the partitions measured (effective reaction size) | **E** | qPCR 50 µL,  RainDrop® dPCR ~50 µL  QX200™ ~15 µL |
| Partition volume variance/standard deviation | D |  |
| Comprehensive details and appropriate use of controls | **E** | Supplementary Information section 4 |
| Manufacturer of dPCR instrument | **E** | Supplementary Information section 4 |
| **qPCR/dPCR VALIDATION** |  |  |
| Optimisation data for the assay | D |  |
| Specificity (when measuring rare mutations, pathogen sequences etc.) Gel, sequence, melt, or digest | **E** | Specificity determined *in silico* using NCBI BLAST |
| If multiplexing, comparison with singleplex assays | **E** | qPCR equivalent sensitivity of multiplex & singleplex |
| qPCR SYBR Green I: Cq of the NTC | **E** | N/A |
| qPCR standard curves with slope and y-intercept | **E** | Available on request |
| PCR efficiency calculated from slope | **E** | qPCR efficiency approximately 93% |
| Confidence interval for PCR efficiency or standard error | D |  |
| r2 of standard curve | **E** | qPCR: R^2^ approximately 0.99 |
| qPCR linear dynamic range | **E** | >5 logs_10_ |
| Cq variation at lower limit | **E** | Not tested |
| Confidence intervals throughout range | D |  |
| qPCR: Evidence for limit of detection | **E** | Probit analysis 15 HIV DNA copies per million cells (95% detection) see Table S5. |
| Limit of detection of dPCR calibration control | D |  |
| **DATA ANALYSIS** |  |  |
| Average copies per partition (λ or equivalent ) | **E** | Materials and Methods |
| Cq method determination | **E** | Manual threshold setting in Applied Biosystems Sequence Detection Software v1.4 |
| qPCR/dPCR analysis program (source, version) | **E** | Supplementary Information section 4 |
| Outlier identification and disposition | **E** | N/A |
| Results of NTCs | **E** | Supplementary Information section 5 |
| Examples of positive(s) and negative experimental results as supplemental data | **E** | Supplementary Figures S1 & S2 |
| Where appropriate, justification of number and choice of reference genes | **E** | N/A |
| Where appropriate, description of normalisation method | **E** | Materials and Methods; Supplementary Information |
| Number and concordance of biological replicates | D |  |
| Number and stage (RT or qPCR) of technical replicates | **E** | Materials and Methods; Supplementary Information |
| Repeatability (intra-assay variation) | **E** | Fig. 2a |
| Reproducibility (inter-assay/user/lab etc. variation ) | D |  |
| Experimental variance or confidence interval | **E** | Coefficient of Variation CV%  qPCR (excluding extraction step): ~ 14%  dPCR (including extraction step)  RainDrop**®**: <10%  QX200™: ≤5% |
| Statistical methods used for analysis | **E** | Materials and Methods |
| Data submission using RDML | D |  |
|  |  |  |
| * Assessing the absence of DNA using a no RT assay is essential when first extracting RNA. Once the sample has been validated as RDNA-free, inclusion of a no-RT control is desirable, but no longer essential. | | |
|  |  |  |
| ** Disclosure of the primer and probe sequence is highly desirable and strongly encouraged. However, since not all commercial pre-designed assay vendors provide this information when it is not available assay context sequences must be submitted (Bustin et al. Clin Chem. 2011 Jun;57(6):919-21.) | | |
|  | | |

## 4. qPCR/digital PCR experimental protocols

**qPCR analysis of clinical samples**

qPCR analysis of 18 PBMC sample extracts was performed using an Applied Biosystems® 7500 Real-Time PCR System. Experiments were implemented in accordance with the MIQE guidelines, [^3^](#_ENREF_3) (Table S2, Supplementary Information). To prepare a qPCR calibration curve consisting of ~50,000 to ~5 HIV DNA copies per reaction (assuming 1 HIV DNA copy per 8E5 cell), DNA extracted from the 8E5 cell line Standard 1, (DNA concentration initially established by Qubit fluorometric quantitation; ThermoFisher Scientific Inc.), was serially diluted using a tenfold dilution series in 5 µg/mL carrier RNA (Qiagen) dissolved in nuclease-free water. Twenty µL of each clinical sample extract (~ 1.2 µg DNA) was added to a total reaction volume of 50 µL. The reaction mix contained 1x QuantiTect Multiplex PCR Master Mix (with ROX dye) (Qiagen Cat. No. 204543), sterile nuclease-free water and the PDH/HIV LTR-*gag* duplex assay with primer and probe concentrations as detailed in Table S1. Thermocycling conditions were as follows: 15 minutes at 95 °C (DNA polymerase activation step), then 45 cycles of 94 °C for 60 s and 60 °C for 60 s. The serially diluted 8E5 calibrator (Standard 1) simultaneously provided standard curves for both PDH and HIV-1 DNA using Applied Biosystems SDS v1.4 analysis software. Quantification of the PDH gene, present as a single copy per haploid genome, was used to estimate the total number of cell equivalents in each amplification reaction. HIV DNA copies per million cells were calculated by dividing the HIV DNA copy number by the number of cells and multiplying by one million. Probit analysis (Arcus Quickstat Biomedical v1.0) demonstrated that the 95% detection limit of the qPCR assay in this duplex format is approximately 15 HIV DNA copies per million cells (Table S5).

**Digital PCR basic protocol**

Duplex format dPCR experiments were implemented in accordance with the dMIQE guidelines (Supplementary Information) [^4^](#_ENREF_4). Two dPCR instruments were employed during the study; the RainDrop® Digital PCR System (RainDance Technologies) was used to measure the clinical samples and 8E5 extracts, and the QX200™ Droplet Digital PCR System (BioRad) was used to measure the 8E5 extracts only. No template controls (NTCs) were included in all experiments (Table S5)

For The RainDrop® instrument, 5.5 µL template was added to a total master reaction volume of 55 µL containing 1x TaqMan® Genotyping Master Mix (Thermo Fisher), 1x droplet stabiliser (RainDance Technologies), sterile nuclease-free water (Ambion) and the chosen primer assay duplex. 50 µL of reaction mix was pipetted into a RainDrop® Source chip and droplets were generated as described [^5^](#_ENREF_5) and cycled on a Tetrad PTC-225 Thermal Cycler (MJ Research). Thermal cycling conditions were: 10 minutes at 95 °C, 45 cycles of 95 °C for 15s and 60 °C for 60s, 10 minutes at 98 °C and a 10 minute hold at 12 °C. A ramp rate of 0.5 °C/sec was maintained for all stages of thermal cycling. Following PCR amplification, droplets were read on the RainDrop® Sense as described [^5^](#_ENREF_5) and data analysed with RainDrop® Analyst II.

For the QX200™ Droplet Digital PCR System varying volumes of template (see below) were added to a master reaction volume of 22 µL containing 1X ddPCR Supermix for Probes without dUTP (BioRad), sterile nuclease-free water and the selected primer assay duplex. 20 µL of this was pipetted into the sample well of a DG8 cartridge and droplets generated as previously described [^6^](#_ENREF_6). Thermocycling conditions were as follows: 10 minutes at 95 °C, 40 cycles of 94 °C for 30 s and 60 °C for 1 min, followed by 98 °C for 10 min and a 4 °C hold. The ramp rate for each step was 2 °C/s. Droplets were read using the QX200™ Droplet Reader and the data analysed using QuantaSoft version 1.6.6.0320.

## Supplementary results

## 5. Number of HIV DNA copies measured in 18 clinical samples using qPCR and dPCR

Table S3. Raw HIV DNA copies per reaction for qPCR and RainDrop® dPCR.

|  | **Raw HIV DNA copies per reaction** | |
| --- | --- | --- |
|  | **qPCR** | **RainDrop®** |
| **Reaction volume (µL)** | 50 | ~50 |
| **Sample volume (µL)** | 20 | ~5 |
| Sample 1 | 22 | 0 |
| Sample 2 | 204 | 7 |
| Sample 3 | 263 | 31 |
| Sample 4 | 2041 | 77 |
| Sample 5 | 2824 | 110 |
| Sample 6 | 2 | 0 |
| Sample 7 | 11 | 2 |
| Sample 8 | 358 | 20 |
| Sample 9 | 16 | 3 |
| Sample 10 | 3 | 0 |
| Sample 11 | 1569 | 116 |
| Sample 12 | 109 | 4 |
| Sample 13 | 171 | 12 |
| Sample 14 | 52 | 4 |
| Sample 15 | 2497 | 81 |
| Sample 16 | 108 | 12 |
| Sample 17 | 392 | 16 |
| Sample 18 | 185 | 16 |

## 6. Assessing dPCR false positive detection of HIV DNA

Table S4. Details of negative controls included in all dPCR experiments.

| **Instrument** | **Number of experiments** | **Total number of NTCs** | | **Number of positives** |
| --- | --- | --- | --- | --- |
| **RainDrop®** | 12 | 12 | 0 | |
| **QX200™** | 2 | 7 | 0 | |

Figure S1. Examples of positive and negative results for the RainDrop® and QX200™ experiments on the 8E5 DNA standards. 2-dimensional amplification plots of 8E5 Standard 3 (passage 2) analysed using (**A**) the RainDrop® and (**C**) the QX200™, and the corresponding respective NTCs (**B**) and (**D**).


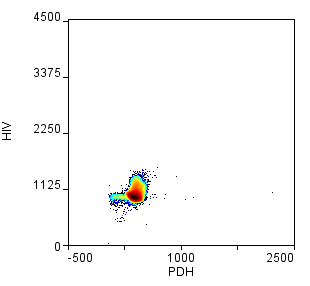

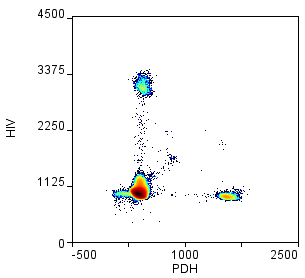


**A**

**B**


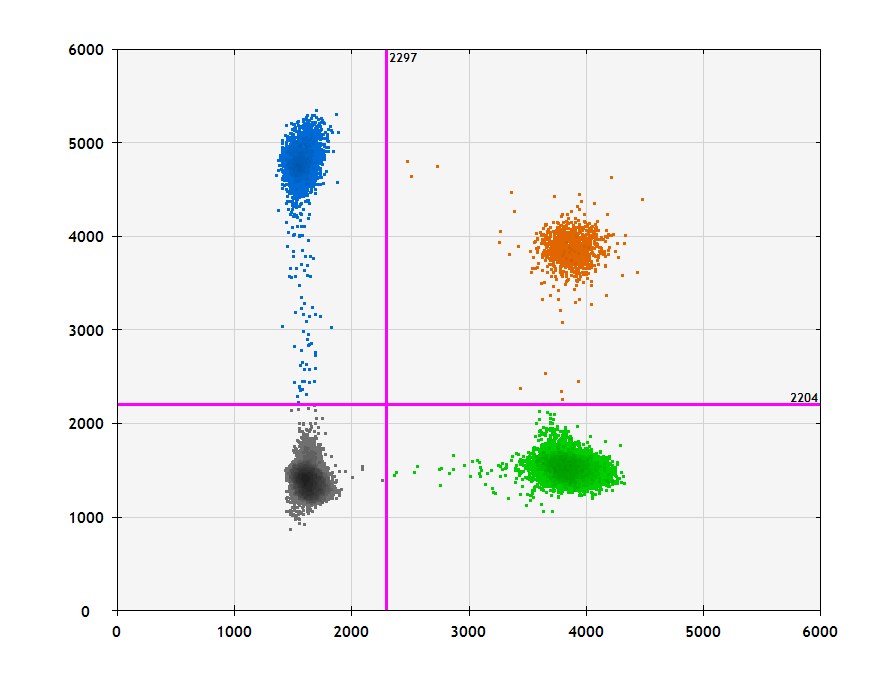

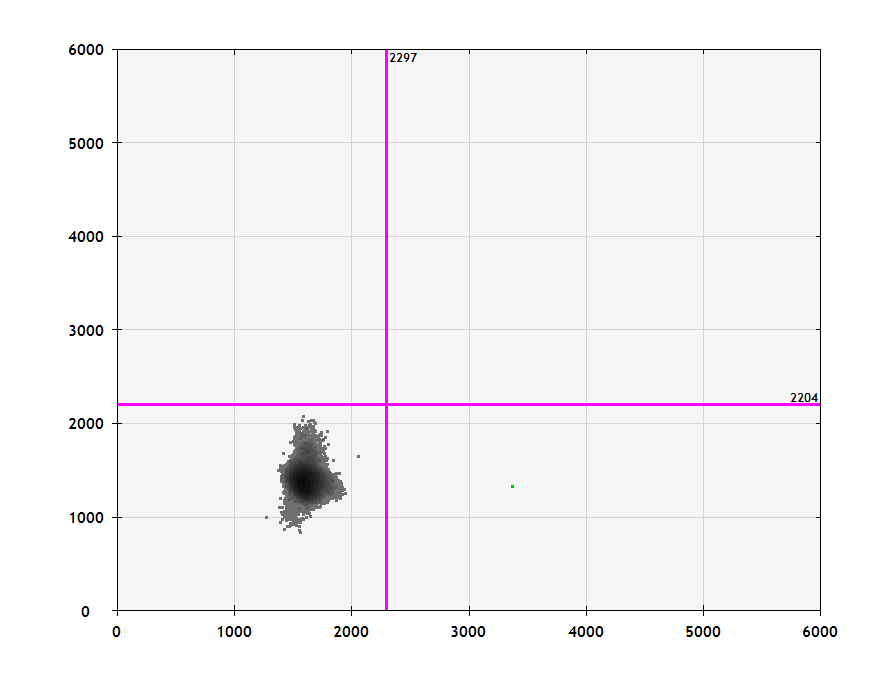

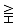

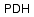

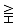

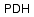


**C**

**D**

## 7. Digital PCR analysis of clinical samples

Figure S2. Examples of 2-dimensional RainDrop® plots for clinical samples containing high (**A**) and low (**B**) concentrations of HIV DNA, and NTCs (**C**)


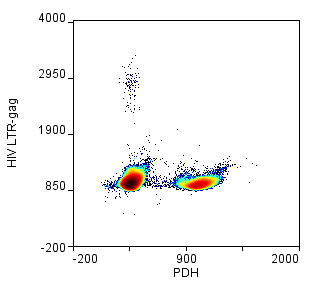

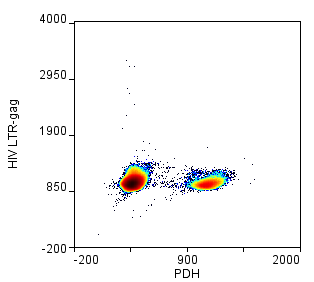


**A**

**B**


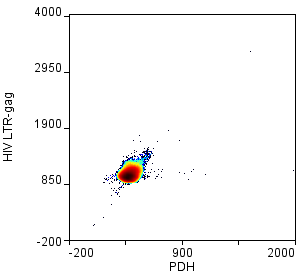


**C**

## 8. Bland-Altman analysis comparing dPCR with qPCR

Figure S3. Bland-Altman analysis of qPCR and dPCR (log_10_ HIV DNA per million cells) data to determine whether the magnitude of the difference between the methods was dependent on HIV concentration. Analysis comparing the difference between methods (qPCR – dPCR) against the mean result of the two methods for a given sample.

Red triangles represent data using qPCR quantities calculated assuming one HIV DNA copy per 8E5 cell and blue triangles, data using qPCR quantities calculated assuming 0.6 HIV DNA copy per 8E5 cell as determined by dPCR. Linear regression analysis demonstrated that neither data set generated regression coefficients that differed significantly from 0 (p = >0.06).

## 9. Probit analysis of qPCR HIV assay

Table S5. Details of probit data for the HIV qPCR assay

| HIV copies per million cells | Number of trials | Number of positives |
| --- | --- | --- |
| 20 | 12 | 12 |
| 10 | 11 | 10 |
| 5 | 12 | 8 |
| 2.5 | 12 | 4 |
| 1.25 | 15 | 4 |
| 0 | 30 | 0 |

References

1 Devonshire, A. S. *et al.* Towards standardisation of cell-free DNA measurement in plasma: controls for extraction efficiency, fragment size bias and quantification. *Analytical and bioanalytical chemistry* **406**, 6499-6512, doi:10.1007/s00216-014-7835-3 (2014).

2 Eriksson, S. *et al.* Comparative analysis of measures of viral reservoirs in HIV-1 eradication studies. *PLoS pathogens* **9**, e1003174, doi:10.1371/journal.ppat.1003174 (2013).

3 Bustin, S. A. *et al.* The MIQE guidelines: minimum information for publication of quantitative real-time PCR experiments. *Clinical chemistry* **55**, 611-622, doi:10.1373/clinchem.2008.112797 (2009).

4 Huggett, J. F. *et al.* The digital MIQE guidelines: Minimum Information for Publication of Quantitative Digital PCR Experiments. *Clinical chemistry* **59**, 892-902, doi:10.1373/clinchem.2013.206375 (2013).

5 Milbury, C. A. *et al.* Determining lower limits of detection of digital PCR assays for cancer-related gene mutations. *Biomolecular Detection and Quantification* **1**, 8-22, doi:10.1016/j.bdq.2014.08.001 (2014).

6 Devonshire, A. S. *et al.* Highly reproducible absolute quantification of Mycobacterium tuberculosis complex by digital PCR. *Analytical chemistry* **87**, 3706-3713, doi:10.1021/ac5041617 (2015).
